# Useful Biomarkers of Metabolic Syndrome

**DOI:** 10.3390/ijerph192215003

**Published:** 2022-11-15

**Authors:** Younghye Cho, Sang Yeoup Lee

**Affiliations:** 1 Department of Family Medicine and Biomedical Research Institute, Yangsan Hospital, Pusan National University, Yangsan 50612, Republic of Korea; 2 Department of Medical Education, School of Medicine, Pusan National University, Yangsan 50612, Republic of Korea; 3 Integrated Research Institute for Natural Ingredients and Functional Foods, Yangsan 50612, Republic of Korea

## 1. Introduction

The Special Issue call for papers on “Metabolic syndrome and its association with biomarkers” was proposed to present research on various markers for pathophysiology and the early detection of metabolic syndrome (MetS).

MetS is a cluster of risk factors for developing heart disease, stroke, and type 2 diabetes [1]. Visceral adiposity and insulin resistance are crucial mechanisms of MetS [2,3].

After the WHO group defined MetS in 1998 [4], efforts to unify the definition of MetS have been continued in accredited organizations. However, various diagnostic criteria are still being used in research [5,6,7]. MetS is usually diagnosed when three or more risk factors are present among abdominal obesity, high blood pressure, hypertriglyceridemia, hyperglycemia, and low high-density lipoprotein cholesterol levels [7].

Numerous biomarkers have been discovered to better understand the pathophysiology and detect MetS early, including adipokines and inflammatory markers. We would like to present clinically useful Mets biomarkers with sufficient evidence.

## 2. Useful Biomarkers of Metabolic Syndrome

### 2.1. Adipokines

Leptin is a hormone that regulates energy metabolism by suppressing food intake and increasing energy expenditure, and is known to be related to abdominal obesity and insulin resistance, which are essential factors for MetS [8]. In large-scale cross-sectional studies, leptin levels were significantly positively associated with MetS [9,10,11]. The significant association between leptin and MetS presented after adjusting covariates, including age, insulin resistance marker, and body mass index (BMI) [9]. In addition, high leptin levels have been suggested as a predictive marker of developing MetS in prospective studies [12,13,14].

Adiponectin improves glucose metabolism and regulates dietary intake and energy expenditure [15]. Low serum adiponectin levels were associated with MetS in many cross-sectional studies [16,17]. In a study including Japanese adults, an adiponectin cutoff value of 4 μg/mL identified most subjects with MetS [17]. Prospective studies suggest the usefulness of adiponectin in predicting Met [18,19]. After a median follow-up of 9.4 years, 1134 healthy participants with low levels of adiponectin (quartile 1) at baseline had an approximately three-fold increased risk of developing MetS compared to those with high levels (quartile 4). Participants who decreased adiponectin levels during follow-up had more than a four-fold increased risk of MetS after multivariate adjustment [19].

High-molecular-weight (HMW) adiponectin, the active form of adiponectin, is a more useful marker than total adiponectin for predicting developing MetS [20]. Low HMW adiponectin levels (≤2.65 μg/mL) were associated with progression to MetS.

Since leptin and adiponectin are independent factors of MetS and have opposing functions in fat metabolism, the leptin to adiponectin (LA) ratio is suggested as a better marker for MetS than adiponectin or leptin alone [21]. A recent prospective study evaluating the association of leptin, adiponectin, and LA rates with future risk of MetS in middle-aged and elderly Koreans showed that leptin, adiponectin levels, and LA rates could be helpful to biomarkers for predicting future MetS incidence [14].

### 2.2. Inflammatory and Oxidative Stress Biomarkers

C-reactive protein (CRP) is a non-specific biomarker commonly used in the evaluation of disease activity, diagnosis, and management of infection, as well as in the differential diagnosis or classification of inflammatory diseases in inflammatory diseases such as rheumatic diseases [22]. Elevated CRP is associated with metabolic disease, including dyslipidemia, diabetes, and metabolic syndrome [23]. In a cross-sectional study in Germany, the age-adjusted geometric means of CRP concentrations in subjects grouped according to the presence of 0–1, 2–3, and 4–5 features of MetS were 1.11, 1.27, and 2.16 mg/L, respectively, with a statistically significant trend [24]. A prospective study evaluating interrelationships between CRP, MetS, and incident cardiovascular events among 14,719 healthy women who were followed up over 8-year follow-up showed CRP works as a prognostic factor on subsequent risk at all severity levels of MetS [25]. In this study, the cutoff point for CRP to differentiate between high-risk and low-risk groups was 3 mg/L. Many studies demonstrate that CRP can be used as a helpful biomarker in MetS.

Ferritin is a vital iron storage protein that regulates iron homeostasis and can reflect the degree of acute and chronic inflammation [26]. A cross-sectional study including 18,581 men showed that the risk of MetS increased about two times in the highest ferritin quartile (≥212.8 ng/mL) compared to the lowest ferritin quartile (<107.3 ng/mL) [27]. Another study of postmenopausal women showed that the OR of the highest ferritin quartile (≥86.0 ng/mL) for MetS was about two times, compared to the lowest quartile (≤36.3 ng/mL), after adjustment for age, smoking, alcohol intake, and regular exercise [28]. The usefulness of ferritin as a biomarker of MetS also showed consistent results in prospective studies [29]. A meta-analysis of 15 observational studies concluded that increased ferritin levels are independently and positively associated with the presence of MetS [30].

Gamma-glutamyltransferase (GGT) is a ubiquitous enzyme in human tissues, including the kidney, pancreas, liver, spleen, heart, and brain, that recycles precursors to the antioxidant and metabolic substrate glutathione (GSH). GGT has been studied as a predictor of MetS, diabetes, hypertension, and stroke risk, and used as a biomarker to evaluate alcohol consumption and hepatobiliary disease [31,32]. A cross-sectional analysis suggested the cutoff value of GGT for MetS was 27 IU/L for men and 17 IU/L for women [33]. Increased serum GGT could predict the onset of metabolic syndrome and the occurrence of CVD and death above and beyond traditional cardiac risk factors, including CRP. The highest GGT quartile experienced a 67% increase in CVD incidence [33].

Uric acid is the end metabolite produced by the breakdown of purines, which are chemicals that enter the bloodstream during the digestion of foods or from the normal breakdown of some of the body’s cells. High uric acid is positively associated with obesity, MetS, type 2 diabetes, and CVDs [34]. In a cross-sectional study using a nationally representative sample of US adults, the prevalence of MetS was 18.9% for uric acid levels less than 6 mg/dL, 59.7% for uric acid levels from 8–8.9 mg/dL, and 70.7% for uric acid levels of 10 mg/dL or greater, and the increasing trends persisted in subgroups adjusted by sex, age group, alcohol intake, body mass index, hypertension, and diabetes [35]. A prospective cohort study including the Korean adult population showed that a high uric acid level could be a positive predictor for MetS for a 2.6-year follow-up period [36]. A dose–response meta-analysis of a prospective study suggested that each 1 mg/dL increase in serum uric acid was linearly associated with MetS risk (RR, 1.30; 95% CI, 1.22–1.38), suggesting that uric acid has proven to be a useful biomarker [37].
